# A multivariate analysis on the comparison of raw notoginseng (*Sanqi*) and its granule products by thin-layer chromatography and ultra-performance liquid chromatography

**DOI:** 10.1186/s13020-015-0040-2

**Published:** 2015-06-06

**Authors:** Xian Zhou, Valentina Razmovski-Naumovski, Kelvin Chan

**Affiliations:** The National Institute of Complementary Medicine, University of Western Sydney, Locked Bag 1797, Penrith, NSW 2751 Australia; Faculty of Pharmacy, The University of Sydney, Sydney, Australia

## Abstract

**Background:**

Granule products produced from medicinal herbs are gaining popularity. However, there have been few studies comparing the quality or efficacy of granules with those of herbal formulations. This study aims to compare commercially available notoginseng (*Sanqi* in Chinese) in both raw and granule forms by thin layer chromatography (TLC) and ultra-performance liquid chromatography with photodiode array detection (UPLC-PDA) using multivariate analysis.

**Methods:**

Aqueous extracts of the raw herb (collected from six different sources in China) and granule products (purchased in China, Taiwan and Australia) were re-extracted with methanol to remove water-soluble excipients. Five compounds (ginsenosides Rg1, Rg2, Rd and Rb1 and notoginsenoside NR1) in the methanolic extracts were quantified by TLC and UPLC-PDA. Multivariate statistical analysis using hierarchical component analysis (HCA) and principal component analysis (PCA) was used to determine the similarities between the granule products and raw herbs. A 2,2′-azino-bis(3-ethylbenzothiazoline-6-sulfonic acid) (ABTS) assay was used to measure the antioxidant capacities of the extracts.

**Results:**

HCA and PCA of the TLC analysis clustered the granule products into one group. By UPLC analysis, the raw herbs and two of the granule products (G7 and G12) were allocated into Group 1 and the rest of the granule products into Group 2. The contents of the five marker compounds in Group 1 were higher than Group 2 and also exhibited stronger ABTS activity (*P* = 0.005). By Pearson correlation, the contents of the five compounds in the samples were positively and significantly correlated to their antioxidant activities.

**Conclusions:**

UPLC was more efficient than TLC for the simultaneous determination of the five major compounds in *Sanqi* products in terms of linearity, higher sensitivity and repeatability. The statistical analysis of the samples by HCA and PCA revealed that the contents of the marker compounds were significantly higher in the raw herb group than the granule group.

**Electronic supplementary material:**

The online version of this article (doi:10.1186/s13020-015-0040-2) contains supplementary material, which is available to authorized users.

## Background

The use of herbal preparations as pharmaceutical products is considered convenient, portable and consistent [1]. Granule products of herbal preparations are increasingly popular among consumers. Granule products for use in traditional Chinese medicine are prepared by concentrating a herbal extract to a dry powder, and then adding excipients such as starch, dextrins, lactose and soluble fibres [1]. As with pharmaceutical preparations, these products must meet minimum quality, safety and efficacy requirements, especially testing the pharmacological efficacy of granule products against decoctions prepared by boiling medicinal herbs in water. Non-standardised manufacturing and quality control procedures would inadvertently introduce clinical inconsistency in dosages for consumers. Currently, there are no established regulatory guidelines for the standardisation of granule products, and few studies have compared the granule’s chemical profile to that of the raw material. Thus, the quality assurance/control of granule products is a critical issue for their future development.

Thin layer chromatography (TLC) and high performance liquid chromatography are standard industry methods for the quality control of herbal products [2–4]. TLC has been used extensively in industry because of its simplicity, low cost and versatility for simultaneous analysis of multiple samples [5]. In the last decade, ultra-performance liquid chromatography (UPLC) has become the preferred method for the analysis of herbal products because of its sensitivity and high resolution for the quantification of active components [5–7].

Multivariate statistical analyses such as hierarchical component analysis (HCA) and principal component analysis (PCA) are employed to compare multiple samples [6]. HCA classifies samples into clusters according to their similarities or differences. PCA uses a linear mathematical algorithm to derive principal components (PCs), and assesses how certain combinations of key factors account for differences between samples [8].

Notoginseng (*Sanqi*) is the root and rhizome of *Panax notoginseng* (Burk.) F. H. Chen. There is a large market for notoginseng granules in China, with data from the China Food and Drug Administration showing more than 50 granule products are currently manufactured [9]. These products are also widely available in Australia and other Western countries. Therefore, *Sanqi* was chosen as an example for the quality control of granule products. *Sanqi* is commonly used for the management of cardiovascular complications [10] because of its antioxidant, antiplatelet [11], haemostatic [12] and fibrinolytic activity [13]. The plant-based antioxidant effects were involved in the prevention and treatment of cardiovascular diseases [14, 15]. The antioxidant activities of the extracts of raw notoginseng and its saponin compounds have been well studied [10]. The antioxidant capacities of notoginseng products can be measured using the simple 2,2′-azino-bis(3-ethylbenzothiazoline-6-sulfonic acid (ABTS) assay, and the results used to assess the claimed bioactivity. However, no studies have assessed the antioxidant effects of granule formulations in comparison with the raw herb.

This study aims to compare commercially available notoginseng (*Sanqi*) in both raw and granule forms by TLC and UPLC-photodiode array (PDA) using multivariate analysis. In this study, the ginsenosides Rg1, Rg2, Rd and Rb1 and notoginseng R1 (NR1) (Additional file 1) were qualitatively and quantitatively evaluated in raw notoginseng and granule products using TLC. The results were compared with those from UPLC with PDA detection, and the quantitative data were analysed by HCA and PCA. The ABTS assay was used to determine the antioxidant capacities of the samples and examine any correlation of this to the amount of the marker compounds. The results from this *in vitro* chemical assay, when correlated to the chemical profiles of the samples, will provide further information about the quality of the notoginseng products.

## Methods

### Chemicals and plant materials

Raw notoginseng (R1-R6) was collected from six different sources in China and twelve herbal granule products (G1-G12) were purchased in China, Taiwan and Australia (Additional file 2). The raw herb samples were authenticated by Professor Si-bao Chen (Department of Applied Biology and Chemical Technology, Hong Kong Polytechnic University, Hong Kong, China) according to the Hong Kong Materia Medica Standards and Pharmacopoeia of the People’s Republic of China 2010 (PPRC). A voucher specimen of each sample was deposited in the National Institute of Complementary Medicine (NICM), University of Western Sydney. Product names have been omitted due to the absence of consent for disclosure. The five marker compounds, notoginseng NR1 and ginsenosides Rg1, Rb1, Rd and Rg2, were purchased from Chengdu Biopurify Phytochemicals Ltd. (Chengdu, China; purity >98 %). HPLC grade acetonitrile and methanol were purchased from Thermo Fisher Scientific (Waltham, MA, USA). Analytical grade ethyl acetate, sulfuric acid and chloroform were purchased from Ajax Finechem (Sydney, Australia).

### Sample preparation

The manufacturing process for granule products was considered an industrial-scale reproduction of a water decoction. In this study, 1 g of a ground raw herb sample (30-mesh size) was refluxed in 30 mL of boiling water three times for 30 min. The aqueous extracts were combined then centrifuged at 672 × *g* for 5 min and evaporated to dryness. Each aqueous raw herb extract and 1 g of each granule sample was sonicated in 10 mL of methanol for 30 min (done three times), followed by centrifugation at 672 × *g* for 5 min. The supernatants were collected and evaporated to dryness at 60 °C under vacuum. This procedure separated the water-soluble excipients from the granule samples, and allowed for comparison of the two products as methanol extracts (Additional file 3). The dry residue was weighed and re-dissolved in a minimum volume of methanol for further analysis.

### Preparation of standard solutions

Stock standard solutions of the reference marker compounds Rg1, Rg2, Rd, Rb1 and NR1 (2 mg/mL) were prepared in methanol and stored at 4 °C. Working standard solutions for calibration at five different concentrations were freshly prepared by dilution of the stock solutions. The concentration ranges of the calibration curves for TLC and UPLC were 0.05–1 mg/mL and 0.0125–1 mg/mL, respectively.

### Instrumentation and chromatographic conditions

#### TLC

A TLC kit (CAMAG Chemie-Erzeugnisse & Adsorptionstechnik AG, Muttenz, Switzerland) containing a Linomat 5 automatic applicator with 100-μL syringes and a software-linked (winCATs ver.1.3.0 system) imaging device was used for TLC. TLC plates were evaluated using a CAMAG Scanner 3 with visible light, 366 nm light and 254 nm light, and a camera (Canon PSG × digital camera). The TLC plates were silica gel 60 F_254_ plate (20 cm × 20 cm) (Merck KGaA, Darmstadt, Germany), and each plate was cut into 10 cm × 10 cm squares before use. The application position was 10 mm from the lower edge of the TLC plate. All samples were applied according to the following settings: 8-mm band width, 2-mm space between tracks, and eight tracks on each plate. Standards and samples (6 μL) were loaded onto the TLC plates. All remaining measurement parameters were default settings. CAMAG Twin Trough chambers (10 × 10 cm) with a stainless steel lid were used for the development of the plates with a mobile phase of chloroform–ethyl acetate–methanol–water (15:40:22:9, v/v/v/v) as described previously [16]. The chamber was kept in a fume hood at an ambient temperature of 20 °C. The plate was developed vertically from the lower edge to 80 mm. After development, the plate was air-dried for 10 min before derivatisation. Ginsenosides have weak absorption at low UV wavelengths as they do not possess a strong chromophore [17]. Therefore, the plates were dipped into ice cold sulfuric acid (10 % sulfuric acid in iced methanol) in a CAMAG chamber tank. The plates were then air-dried for 10 min, and heated at 100 °C in an oven for 5 min. The analytes were quantified with the CAMAG Scanner 3 using a D2&W lamp set at 366 nm, with 20-mm/s scanning speed. A CAMAG Reprostar 3 with winCATs software was used to analyse the derivatised plates.

#### UPLC–PDA

UPLC-PDA analyses were performed using a Waters ACQUITY UPLC™ system (Waters, Milford, USA), equipped with quaternary solvent manager (ACQ-QSM), quaternary pump, sampler manager FTN (ACQ-FTN), column compartment, PDA detector (ACQ-PDA), and connected to Waters Empower 3 software. UPLC separations were carried out using an Acquity UPLC BEH C_18_ column (150 mm × 2.1 mm, 1.7 μm) with an attached pre-column (2.1 mm × 5 mm, 1.7 μm) (Waters). The column and sample temperature were kept at 20 °C and 4 °C, respectively. The initial mobile phase consisted of water (A)-acetonitrile (B) (82:18, v/v). Gradient conditions were based on a modification of a method described previously [18], with a gradient elution as follows: 0–5.5 min, 18–19 % B; 5.5–6.0 min, 19–31 % B; 6.0–9.5 min, 31–35 % B; 9.5–12.0 min, 35–56 % B, 100 % B for 6 min. The column was reconditioned isocratically with 18 % B for 7 min. The flow rate was 0.30 mL min^−1^ and the injection volume was 1 μL. The detection wavelength was 203 nm. All solutions were filtered using 0.2-μm polytetrafluoroethylene membrane filters before injection. The total run time for the analysis was 25 min. The identification of compounds in the samples was carried out by comparison of UV spectra and retention times.

#### LC-MS

The purity of the references was verified by liquid chromatography-mass spectrometry (LC-MS). LC-MS experiments were performed on a Waters Acquity Xevo TQ triple quadruple mass spectrometer coupled to a binary pump, PDA detector and an autosampler (Waters, Milford, USA). Mass spectra were acquired in negative electrospray ionisation (ESI) mode with a mass range of *m*/*z* 100–1200. The data were analysed by MassLynx Mass Spectrometry software (Waters, Milford, USA).

#### Validation procedure

The TLC and UPLC methods were partially validated in terms of linearity and repeatability. Six-point calibration curves were constructed with linear ranges of 0.05–1 mg/mL and 0.0125–1 mg/mL for TLC and UPLC, respectively. Six replicates of the calibration standards were prepared, and each was analysed in triplicate. Regression equations, *y* = a*x* + b, were calculated, where *x* and *y* are the concentration of the reference samples and the peak area, respectively. The LOD and LOQ were calculated according to the equations, LOD = 3.33 × (standard deviation [SD] of *y*-intercept/mean of slope) and LOQ = 10 × (SD of *y*-intercept/mean of slope) [19]. The quantity of each analyte was obtained from the corresponding calibration curve. The relative standard deviation (RSD) was used as a measure of repeatability. The intra-day precision was evaluated by analysing four concentrations of each marker compound three times within a day, and the inter-day reproducibility was examined on three consecutive days.

### ABTS antioxidant assay

A modification of an established procedure [20] was used to estimate the ABTS radical scavenging capacities of the notoginseng extracts. The ABTS radical working solution was prepared by mixing equal volumes of 7 mmol/L ABTS solution with 2.45 mmol/L potassium persulfate solution, and the mixture was left in the dark for 12 to 16 h at room temperature. On the day of analysis, the stock solution was diluted with PBS (pH 7.4) until an initial absorbance value of 0.4 at 730 nm was reached. Diluted ABTS (200 μL) was mixed with 20 μL of the sample or standard and the absorbance reading was taken 5 min after mixing. Trolox (0.045–0.330 mmol/L) was used as the standard. The antioxidant activity was calculated as the concentration of ABTS ^+^ quenched by 1 mmol/L of Trolox. The antioxidant activities of the notoginseng samples were expressed as Trolox equivalents per dry weight (DW) of the sample (mmol L^−1^/g of DW) [21]. The ABTS assay was performed in triplicate.

### Statistical analysis

The results from the granules were converted back to raw herb values, by the ratio specified on the product, to compare the raw herb and granule products. Here, the residue obtained from the granules after the methanol extraction was assumed to be equivalent to the raw herb water extract without excipients. The yields were expressed as the mean ± SD of three extractions. Each of the three extracts from the same sample were analysed three times by TLC and UPLC, with the final quantitative results from TLC and UPLC instrumental analyses expressed as the mean ± SD. Quantitative results were reported as milligrams per grams of the DW of the raw herb (mg/g).

The TLC and UPLC quantitative data were analysed non-parametrically by IBM SPSS Statistics 20 for Windows (SPSS Inc., Chicago, IL, USA) to determine any significant differences in the marker compound contents between the raw herbs and granules. The compound(s) that showed significant differences were assigned as variables for HCA. The data were pretreated by autoscaling. HCA was conducted by the Ward’s method and Euclidean distances. The results were expressed as dendrograms where the length of the branches between samples reflected the degree of similarity between them.

PCA was conducted by XLSTAT (Addinsoft, New York, USA). PCA converted the original variables (five marker compounds) into a new set of linearly uncorrelated factors (PCs). The first and second PCs corresponded to the largest possible variance of the original variables. The results were represented in a biplot (score plot and loading plot), which showed the distribution of the samples and the correlation of the five original variables to the two PCs [8, 22].

The yield and ABTS results were analysed by independent samples *t*-test and non-parametric analysis. Pearson correlation coefficients (r) indicated the strength of the correlation between the contents of the five marker compounds in the samples and their ABTS scavenging activities. These analyses were conducted by SPSS, and *P* < 0.05 was considered statistically significant.

## Results and discussion

### Extraction yield

The average amount of dry residue obtained from each sample is shown in Table 1. The average yields of the raw herbs (R1–R6) were consistent at 21.07 ± 3.39 % of the dry weight of the herb. Independent samples *t*-tests illustrated that, within the raw herb group, the yield from R5 was significantly lower than those from the rest of the raw herbs (*P* = 0.029). By contrast, the average yields of the granule products were variable and ranged from 1.07–18.85 %. The differences in the yield for the granule products could be caused by the varied manufacturing processes used by the companies that produced the granules. For example, extraction procedures, temperature conditions, the type and amount of excipients used, and other factors in the manufacturing processes can affect the quality of the finished product. A comparison of the two groups using non-parametric analysis showed that the yields from the raw herbs were significantly higher than those from the granules (*P* = 0.000). The excipients added to the granules during manufacturing might be slightly soluble in methanol, and may have affected the final calculation of the notoginseng content of the granule product thus, giving a lower concentration for the granule extract. The RSD yields for the raw herbs ranged from 0.19–8.66 %, whereas the RSD yields for the granules ranged from 0.95–23.014 %, indicating that the granule particles were not uniform, even though every granule bottle was shaken before sampling to redistribute the particles.

**Table 1:** The average yields of the raw herbs (R1-R6) and granule (G1-G12) methanol extracts (n ≥ 3)

Samples	Average yield (%)	RSD (%)^a^
R1	24.17	8.65
R2	23.52	4.34
R3	23.26	0.13
R4	19.82	6.71
R5	15.150	1.06
R6	20.50	5.71
G1	3.59	11.75
G2	8.58	7.44
G3	9.17	13.02
G4	3.16	7.39
G5	3.12	22.98
G6	2.02	0.94
G7	12.47	3.37
G8	1.07	9.01
G9	7.62	6.83
G10	7.16	9.66
G11	11.54	9.79
G12	18.85	7.07

*R* raw herb; *G* granule

^a^RSD (%) = 100 × S.D./mean

Some of the granule products did not have the notoginseng content on the product label. One company explained that this information was not provided on the product label because it could vary from batch to batch. This variation could arise from collecting starting materials from different sources. Differences in the raw herbs would affect the amount of residue obtained after processing and extraction. The non-standardised manufacturing processes for granule products could affect the quality of the products and result in efficacy issues for practitioners and consumers.

### Validation of TLC and UPLC method

#### Calibration, linearity, LOQ and LOD

The linear regression equations, coefficient of determination (R^2^), limit of quantification (LOQ), limit of detection (LOD), average intra- and inter-day repeatability (RSD) for each standard were shown in Table 2. The R^2^ values were greater than 0.991 for all the analytes (*P* values corresponding to a R^2^ value of 0.000), showing good linearity for the experimental data for both analytical methods. The LODs for TLC and UPLC of the five marker compounds ranged from 12.60–144.00 μg/mL and 3.31–16.20 μg/mL, respectively. The LOQs for TLC and UPLC of the five marker compounds were 37.90–378.00 and 9.95–48.80 μg/mL, respectively. The LODs and LOQs from UPLC were generally lower than those from TLC (*P* = 0.016 and *P* = 0.032 for LOD and LOQ, respectively), indicating higher sensitivity was achieved with the UPLC method.

**Table 2:** Regression data, detection/quantification limits and precision data for the five compounds determined by UPLC-PDA

Compounds	Regression equation		R^2^		LOD		LOQ		Precision, RSD (%)		Precision, RSD (%)	
					(μg/mL)		(μg/mL)		Intra-day (*n* = 3)		Inter-day (*n* = 3)	
	TLC*	UPLC*	TLC	UPLC	TLC	UPLC	TLC	UPLC	TLC	UPLC	TLC	UPLC
NR1	y = 5752.1x + 796.27^b^	y = 497892x + 1692^a^	0.993	0.994	16.60	3.77	49.80	11.30	3.17	1.95	1.04	4.59
Rb1	y = 15159x-498.43^a^	y = 451285x-8794.1^a^	0.991	0.997	12.60	4.26	37.90	41.70	7.06	1.67	0.92	0.55
Rd	y = 8877.8x + 1092.3^b^	y = 502877x + 4048.3^a^	0.995	0.994	144.00	3.31	431.00	9.95	1.67	0.74	6.92	1.98
Rg1	y = 8095.7x + 1360.3^a^	y = 519908x-3073.3^a^	0.992	0.993	126.00	16.20	378.00	48.80	2.23	3.58	4.68	2.82
Rg2	y = 11839x + 958.33^a^	y = 529865x + 1901.5^a^	0.991	0.998	33.700	5.97	101.00	17.90	0.77	2.18	2.80	0.26

RSD (%) = 100 × S.D./mean; y, peak area; x, the concentration of each reference compound (mg/mL); R^2^, coefficient of determination; LOD, limit of detection (3.33 × (SD of Y-intercept/mean of slope)); LOQ, limit of quantification (10 × (SD of Y-intercept/mean of slope))

^a^n=3

^b^n=4

*All *P* values for the regression equations are = 0.000

#### Precision

Good instrumental and method precision was obtained for both UPLC and TLC. As shown in Table 2, the RSD for the intra- and inter-day precision ranged from 0.768–7.059 % and 0.916–6.916 % for TLC. For UPLC, the corresponding ranges were 0.739–3.579 % and 0.236–4.591 %, respectively.

### Quantification by TLC

The resolution of TLC for the marker compound notoginseng NR1 and ginsenoside Re was not sufficient to quantify these compounds separately (Additional file 4). As the content of Re in notoginseng is reportedly approximately half that of NR1 [23], the total NR1 + Re peak was used for the quantification of NR1 in this study. The contents for the marker compounds detected in the samples were shown in Table 3. The following compounds were below the limit of quantification: Rg2 in samples R5, G10 and G11, and Rd in sample R5. Fluorescence (UV 366 nm) was used for visualisation of the derivatised saponins, and this enhanced their detection compared with the lower absorbance measurement at 254 nm. However, the noise level also increased [17]. Although the TLC profiles of the raw herbs and granule extracts were similar, the contents of the marker compounds were much higher in the raw herbs than in the granules, with the exception of G7 and G12.

**Table 3:** Contents (mg/g, mean ± SD, n = 3) of the five compounds in *Sanqi* samples analysed by TLC and UPLC-PDA

Sample	NR1		Rb1		Rd		Rg1		Rg2	
	TLC	UPLC	TLC	UPLC	TLC	UPLC	TLC	UPLC	TLC	UPLC
R1	31.77 ± 0.80	6.06 ± 0.97	24.54 ± 0.39	17.82 ± 1.38	3.35 ± 2.07	4.02 ± 0.23	58.06 ± 0.57	38.70 ± 1.51	2.51 ± 0.45	1.83 ± 0.12
R2	41.64 ± 1.47	5.48 ± 1.16	27.38 ± 0.36	19.21 ± 2.37	8.36 ± 0.32	7.08 ± 1.54	41.78 ± 0.81	25.38 ± 2.34	4.59 ± 0.24	3.39 ± 0.77
R3	35.64 ± 0.30	6.05 ± 0.92	31.91 ± 0.77	17.78 ± 0.78	5.94 ± 0.88	6.82 ± 0.12	47.89 ± 1.41	33.87 ± 0.93	3.47 ± 0.34	2.56 ± 0.03
R4	27.15 ± 1.09	9.15 ± 0.23	18.23 ± 0.99	21.54 ± 0.23	11.84 ± 0.65	6.95 ± 0.33	53.80 ± 2.65	39.68 ± 1.40	7.57 ± 0.10	2.30 ± 0.11
R5	8.47 ± 0.77	6.20 ± 0.55	6.56 ± 0.39	12.79 ± 0.38	ND	6.00 ± 0.41	45.35 ± 1.62	35.71 ± 0.73	ND	2.38 ± 0.25
R6	3.03 ± 0.04	4.26 ± 0.05	3.73 ± 0.10	16.48 ± 0.77	2.77 ± 0.06	6.90 ± 0.67	10.55 ± 0.19	23.63 ± 0.39	1.87 ± 0.07	3.59 ± 0.06
G1	2.21 ± 0.29	0.84 ± 0.16	2.93 ± 0.06	3.03 ± 0.23	2.17 ± 0.07	0.80 ± 0.03	4.14 ± 0.02	4.00 ± 0.34	0.63 ± 0.04	0.32 ± 0.00
G2	3.77 ± 0.06	1.37 ± 0.04	4.49 ± 0.32	4.97 ± 0.14	1.20 ± 0.26	2.06 ± 0.20	8.83 ± 0.40	5.15 ± 0.10	2.68 ± 0.16	2.72 ± 0.36
G3	9.46 ± 1.48	2.46 ± 0.03	7.34 ± 0.56	9.74 ± 0.09	3.03 ± 2.56	2.50 ± 0.06	12.79 ± 0.89	12.94 ± 0.14	1.56 ± 0.14	0.57 ± 0.00
G4	0.86 ± 0.13	0.37 ± 0.01	1.24 ± 0.14	1.52 ± 0.09	1.28 ± 0.33	0.38 ± 0.01	4.13 ± 0.48	2.14 ± 0.01	0.58 ± 0.03	0.09 ± 0.01
G5	0.40 ± 0.19	0.52 ± 0.01	1.11 ± 0.02	2.22 ± 0.01	0.02 ± 0.00	0.57 ± 0.01	2.13 ± 0.08	2.91 ± 0.04	0.27 ± 0.03	0.09 ± 0.01
G6	2.51 ± 0.05	0.72 ± 0.01	1.40 ± 0.15	2.56 ± 0.02	0.64 ± 0.04	0.93 ± 0.01	5.14 ± 0.02	3.27 ± 0.03	0.26 ± 0.05	0.19 ± 0.01
G7	11.96 ± 1.10	7.86 ± 0.41	16.59 ± 0.18	32.93 ± 2.01	8.57 ± 2.49	9.52 ± 1.53	27.73 ± 0.84	42.95 ± 2.73	3.11 ± 0.88	1.63 ± 0.24
G8	0.02 ± 0.01	0.08 ± 0.00	0.36 ± 0.01	0.42 ± 0.02	0.35 ± 0.01	0.07 ± 0.00	0.58 ± 0.01	0.52 ± 0.01	0.08 ± 0.01	0.08 ± 0.01
G9	5.39 ± 0.05	2.83 ± 0.07	5.31 ± 0.13	10.60 ± 0.43	3.24 ± 0.13	2.47 ± 0.40	13.58 ± 0.49	15.14 ± 0.59	1.55 ± 0.48	0.57 ± 0.09
G10	5.81 ± 0.25	2.72 ± 0.36	5.59 ± 0.14	12.07 ± 0.95	1.48 ± 0.41	2.94 ± 0.19	13.50 ± 0.08	15.73 ± 1.93	ND	0.52 ± 0.12
G11	12.28 ± 0.09	1.16 ± 0.19	11.22 ± 0.33	4.20 ± 0.62	1.26 ± 0.51	1.71 ± 0.57	19.67 ± 0.09	4.65 ± 0.90	ND	1.98 ± 0.03
G12	11.72 ± 0.71	7.51 ± 0.42	12.67 ± 0.03	24.12 ± 0.15	7.58 ± 0.54	7.28 ± 0.17	30.26 ± 0.95	34.88 ± 0.26	2.56 ± 0.08	1.23 ± 0.24

*ND* not detected

### Quantification by UPLC-PDA

**Fig. 1:**
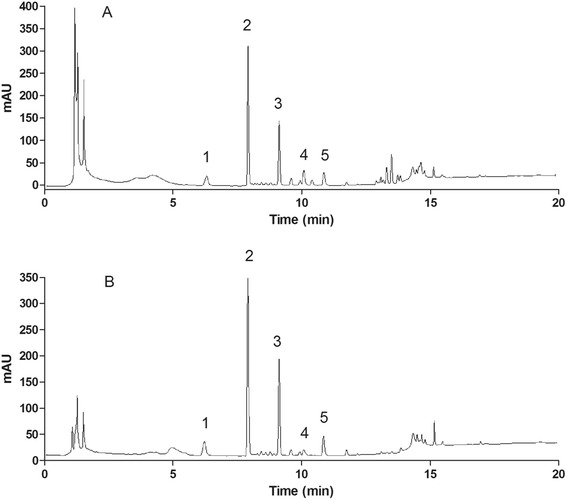
Chromatograms of five ginsenosides analysed by UPLC-PDA. **a** Raw herb extract (R6), **b** Granule extract (G12). (1) NR1, (2) Rg1, (3) Rb1, (4) Rg2 and (5) Rd

Typical UPLC-PDA profiles of the raw notoginseng and granule extracts were shown in Fig. 1. The contents of the marker compounds in the samples were shown in Table 3. After many UPLC runs, ginsenosides Re and Rg1 could not be successfully separated. This was also a problem in previous chromatography studies [24–27]. According to Wan *et al.* [28], the Rg1/Re ratio was 6.19 ± 0.82 in 18 samples of notoginseng from different origins. As the content of Re is much smaller than that of Rg1 and this is a comparative study, the total content of Rg1 and Re was used as the Rg1 content. The low standard deviations of the five standards in the raw herbs and granules showed good repeatability for the quantification (Table 3). The contents of the marker compounds in the granules were substantially lower than those in the raw herb samples, with the exception of G7 and G12. The contents of each of the five compounds were not significantly different within the raw herb samples and within the granule products (*P* > 0.05). However, these two groups were significantly different when the contents were compared (*P =* 0.000). Differences in the contents of the marker compounds in the granules might be related to the variable yields (Table 1) and the different notoginseng contents claimed by the manufacturers.

### Differentiations of raw notoginseng and granules

**Fig. 2:**
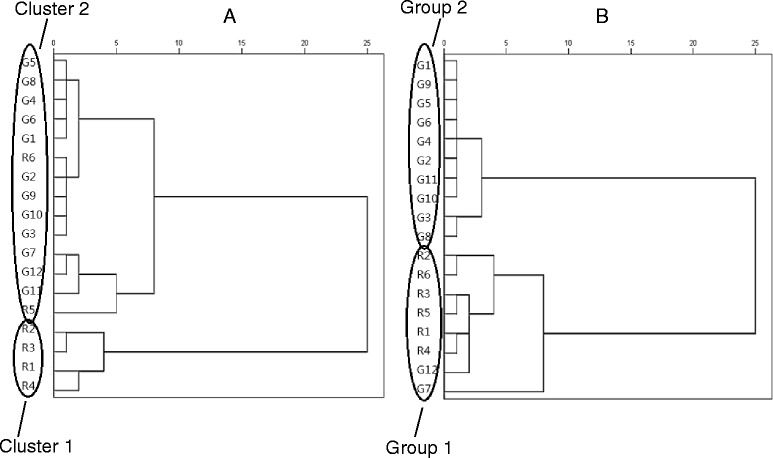
HCA dendrograms of *Sanqi* extracts analysed by (**a**) TLC and (**b**) UPLC. **a** HCA dendrograms for the TLC results divided the raw herb and granule extracts into two main clusters, with raw herb samples R1 to R4 classified into one cluster (Cluster 1), whereas R5 and R6 were grouped with the granules (Cluster 2). **b** The HCA for UPLC grouped granule samples G7 and G12 with the raw herb cluster (Group 1), with the rest of the granules in Group 2

The contents of the five marker compounds were used in HCA analysis for grouping of similar products (Fig. 2), and two groups were found. For TLC, the Cluster 1 included samples R1 to R4, and the Cluster 2 included R5 and R6 and the granule products. The clustering of R5 and R6 by HCA with the granule products could have arisen because the concentrations of some marker compounds in these samples were too low for TLC quantification.

For UPLC, HCA showed a similar pattern, and samples G7 and G12 were grouped with the raw herbs (Group 1) and the rest of the granules as Group 2.

**Fig. 3:**
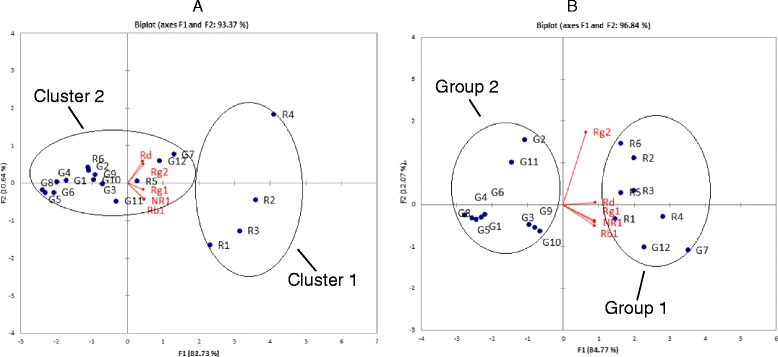
PCA biplot (loading and score plot) of *Sanqi* raw herb and granule extracts analysed by (**a**) TLC and (**b**) UPLC. **a** From the TLC biplot, R1 to R4 demonstrated a higher amount of the compounds (Cluster 1), especially R4 (highest amount of Rd and Rg2). R5 and R6 were closely distributed with the granules (Cluster 2). **b** For UPLC, Group 1 containing the raw herbs, G7 and G12 which possessed higher amounts of the five compounds, whilst Group 2 contained the rest of the granules

PCA was also applied to differentiate samples by displaying them as coordinates in maps based on the contents of the five marker compounds. In the PCA biplot, each point represents an individual sample and the red line represents the contribution of each original variable to the score of two major PCs (Fig. 3). For TLC, PC1 represented up to 82.73 % of the total variance and PC2 (10.64 %) cumulatively explained up to 93.37 % of total variance. The distribution of the samples based on PC1 was due to the variance from all the markers compounds, while the separation of samples by PC2 was based on the contents of Rd and Rg2. The TLC biplot differentiated the raw herbs and granules. Samples R1 to R4 (Cluster 1) showed higher contents of all the marker compounds than the other samples. In particular, R4 had the highest contents of Rd and Rg2. Samples R5 and R6 were plotted close to the granules (Cluster 2).

For UPLC, the biplot showed that PC1 (84.77 %) and PC2 (11.46 %) cumulatively explained up to 96.23 % of total variance. PC1 showed the variance from the original variables, and PC2 was mainly related to Rg2. Based on the two PCs, the PCA biplots for the UPLC results divided the samples into two groups: Group 1 contained the raw herbs and G7 and G12 of the granule products, whilst Group 2 contained the rest of the granule products. This agreed with the UPLC HCA results.

### Current pharmacopoeia standards for notoginseng

In the PPRC, ginsenosides Rg1, Rb1 and notoginseng NR1 are recommended for the quality assessment of the raw herb [29]. The total ginsenosides content (*i.e.*, NR1 + Rg1 + Rb1) in the root and rhizome of notoginseng (methanol extract) should not be less than 5.0 %. However, this guideline does not apply to granules [29]. From the TLC calculations (Table 4), all the raw herb samples (R1–R6) met this minimum requirement, with the total ginsenosides content ranging from 6.0 % to 11.5 %. By contrast, the total ginsenosides content in most of the granules was less than 5.0 % (range 0.1 % to 4.3 %). In the granules, the contents of G7 and G12 were high at 5.6 % and 5.5 %, respectively.

**Table 4:** Pearson correlation between the contents of the compounds analysed by UPLC and the anti-oxidant capacity of the extracts

Components	ABTS
Rg2	0.740^a^
Rd	0.836^a^
Rb1	0.772^a^
NR1	0.769^a^
Rg1	0.768^a^

^a^Correlation is significant at the 0.01 level (2-tailed)

From the UPLC data (Table 3), the raw herb samples (R1–R5) met the minimum requirement of PPRC, with total ginsenosides contents ranging from 5.0 % to 8.2 %. Only sample R6 had a relatively low total ginsenosides content (4.4 %). As with the TLC results, most of the granules contained less than 5.0 % of these three marker compounds, with total ginsenosides contents ranging from 0.1 % to 3.1 %. Again, the contents of G7 and G12 were high at 8.4 % and 6.7 %, respectively. Due to the content differences between raw notoginseng and granules, standards for the granules need to be established and included in future editions of the PPRC.

For comparative purposes, HCA was conducted using the contents of the three marker compounds (NR1, Rg1 and Rb1) specified by the PPRC [29]. From the TLC results, HCA produced clusters that were identical to those derived when using the contents of all five marker compounds studied. However, for the UPLC results, R2 and R6 were grouped with the granules, compared to Group 1 which included all the raw herbs plus G7 and G12 (Additional file 5). These results suggest that more marker compounds (*i.e.*, five rather than three) are required to differentiate raw notoginseng from granule products [29]. A similar finding was reported for the roots of *Pueraria lobata* and *P. thomsonii* and their granule products [30].

### ABTS assay for antioxidants

**Fig. 4:**
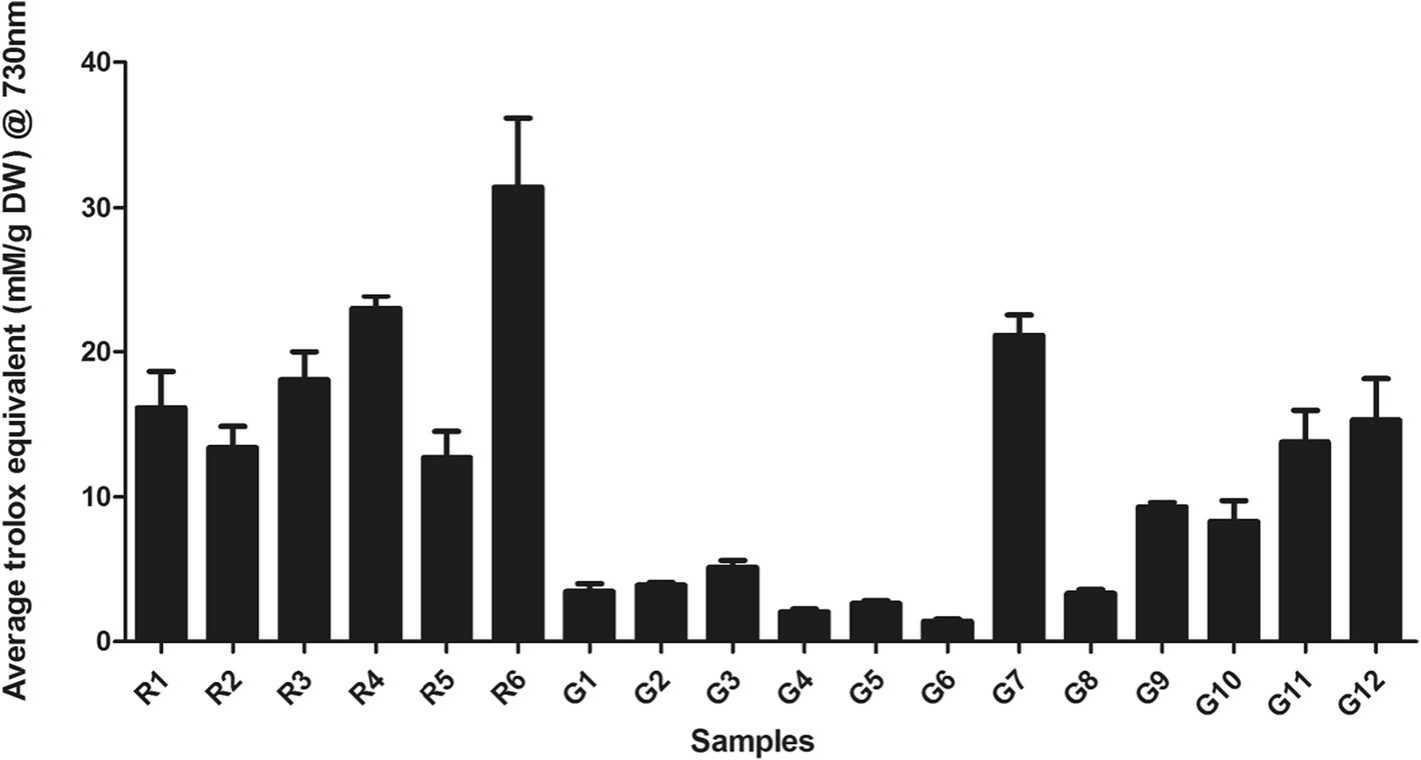
ABTS activity of *Sanqi* raw herb and granule extracts (*n* ≥ 3). Generally, raw herb samples (12.68 – 31.41 mM/g DW) showed a higher ABTS radical scavenging activity than granule samples (1.41 – 21.13 mM/g DW). R6 exhibited the highest anti-oxidant activity among all the samples (31.41 mM/g DW). For the granule samples, G7 and G12 possessed strong radical scavenging activity (21.13 and 15.28 mM/g DW, respectively)

The ABTS assay was conducted at both 730 nm and 410 nm for comparison with the literature, and both wavelengths gave similar results (Fig. 4). The results for the ABTS assay at 730 nm are reported here, as the values are reported as Trolox equivalents and this is measured at 730 nm. R6 exhibited the highest antioxidant activity among all the samples (31.41 mmol L^−1^/g DW). For the granules, both G7 and G12 showed strong radical scavenging activity (21.13 and 15.28 mmol L^−1^/g DW, respectively). Non-parametric analysis showed that the antioxidant capacities of G7 and G12 were similar to R1–R6 (Group 1) (*P* > 0.05). The ABTS radical scavenging activity of Group 1 was significantly higher than that of Group 2 (*P* = 0.005). Pearson correlation was performed to examine the relationship between the quantitative data and radical scavenging capacities. There were significant correlations between the UPLC results for the marker contents and the corresponding antioxidant activities (Table 4).

## Conclusion

UPLC was more efficient than TLC for the simultaneous determination of the five major compounds in *Sanqi* products in terms of linearity, higher sensitivity and repeatability. The statistical analysis of the samples by HCA and PCA revealed that contents of the marker compounds were significantly higher in the raw herb group than the granule group.

## Additional files

Additional file 1:
**Chemical structures of the compounds assessed in the** ***Sanqi*** **samples.** Glc: β-D-glucose; rha: α-L-rhamnose; xyl: β-D-xylose [22]. Additional file 2:
**Information for** ***Sanqi*** **raw herbs and granule samples.** *Sanqi* raw herbs were collected from six different sources in China and twelve herbal granule products were purchased from China, Taiwan and Australia. Additional file 3:
**TLC of methanol extract and residue from the raw herb/granule.** Lane 1: Methanol extract of granule sample 4 (G4); Lane 2: Residue of the methanol extract of G4; Lane 3: Water and methanol extract of the raw herb R1; Lane 4: Residue of water and methanol extract of raw herb. As shown in the figure, Lanes 1 and 3 of the methanol extract contain many bands which represented the marker compounds, whereas Lanes 2 and 4 were absent of the bands. This supported the assumption that methanol efficiently extracted the major compounds. Additional file 4:
**TLC fluorescence image of Sanqi raw herb and granule samples with the standards under UV mode (366 nm).** Key: Lane 1 – Notoginseng NR1; Lane 2 – Ginsenoside Re; Lane 3 – Ginsenoside Rg1; Lane 4 - R6 methanol extract; Lane 5 - G12 methanol extract; Lane 6 -Ginsenoside Rg2; Lane 7 – Ginsenoside Rd; Lane 8– Ginsenoside Rb1. Additional file 5:
**HCA dendograms of Sanqi extracts analysed by (A) TLC and (B) UPLC results using three markers (NR1, Rg1 and Rb1).** For TLC, HCA revealed that the clusters were identical to those derived from the content of the five marker compounds. However, for the HCA result analysed by UPLC, R2 and R6 were grouped with the granules.

## Abbreviations

ABTS: 2,2′-azino-bis(3-ethylbenzothiazoline-6-sulphonic acid)
HCA: Hierarchical component analysis
HPLC: High-performance liquid chromatography
LOD: Limitation of detection
LOQ: Limitation of quantification
NR1: Notoginsenoside R1
PC: Principal component
PCA: Principal component analysis
PPRC: Pharmacopoeia of the People’s Republic of China
Rb1: Ginsenoside Rb1
Rd: Ginsenoside Rd
Rg1: Ginsenoside Rg1
Rg2: Ginsenoside Rg2
RSD: Relative standard deviation
R^2^: Coefficient of determination
TLC: Thin layer chromatography
UPLC-PDA: Ultra-performance liquid chromatography with photo diode array
