# Supplementary material for: A multivariate analysis on the comparison of raw notoginseng (Sanqi) and its granule products by thin-layer chromatography and ultra-performance liquid chromatography
Source: Chin Med. 2015 Jun 6;10:13. doi: 10.1186/s13020-015-0040-2 (PMC4477300; doi:10.1186/s13020-015-0040-2)
Supplement: Additional file 1: — Chemical structures of the compounds assessed in the Sanqi samples. Glc: β-D-glucose; rha: α-L-rhamnose; xyl: β-D-xylose [22]. [file 13020_2015_40_MOESM1_ESM.pdf]

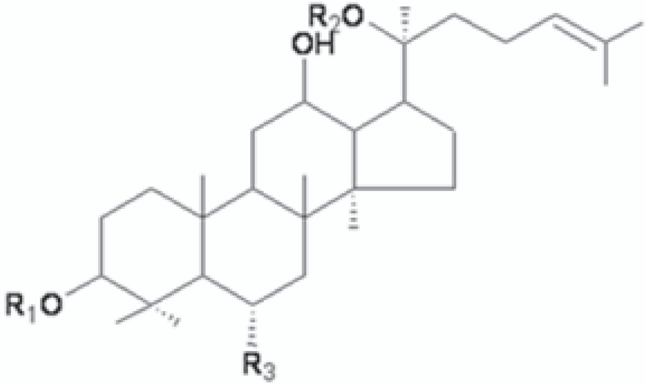

Name	R1	R2	R3	Molecular formula
NR1	-H	-Glc	-Oglc(2-1)xyl	$C_{47}H_{80}O_{18}$
Rb1	-Glc(2-1)glc	-Glc(6-1)glc	-H	$C_{54}H_{92}O_{23}$
Rd	-Glc(2-1)glc	-Glc	-H	$C_{48}H_{82}O_{18}$
Rg1	-H	-Glc	-Oglc	$C_{42}H_{72}O_{14}$
Rg2	-H	-H	-Oglc(2-1)rha	$C_{42}H_{72}O_{14}$
