# Supplementary material for: A multivariate analysis on the comparison of raw notoginseng (Sanqi) and its granule products by thin-layer chromatography and ultra-performance liquid chromatography
Source: Chin Med. 2015 Jun 6;10:13. doi: 10.1186/s13020-015-0040-2 (PMC4477300; doi:10.1186/s13020-015-0040-2)
Supplement: Additional file 2: — Information for Sanqi raw herbs and granule samples. Sanqi raw herbs were collected from six different sources in China and twelve herbal granule products were purchased from China, Taiwan and Australia. [file 13020_2015_40_MOESM2_ESM.pdf]

Sample	Origin	Batch/Lot Number	Ratio (granule to raw herb)
R1	Sichuan, China		
R2	Hong Kong, China		
R3	Yunnan, China		
R4	Hong Kong, China		
R5	Beijing, China		
R6	Jiangsu, China		
G1*	Taiwan	9930	1:5
G2	Guangxi, China	A101175-01	1:5
G3*	Taiwan	5326	1:3
G4*	Jiangsu, China	2009 05	1:5
G5*	China	10105038	1:5
G6*	Sichuan, China	1103073	1:10
G7*	China	20081125	1:1
G8*	Guangdong, China	SQ0610	1:5
G9	Sichuan, China	1203023	1:2
G10	Guangdong, China	1103009N	1:1.3
G11	Beijing, China	110902	1:1
G12	Shenzhen, China	1201001S	2:1.5

*Available in Australia; Key: R: raw herb, G: granule
