# Supplementary material for: A multivariate analysis on the comparison of raw notoginseng (Sanqi) and its granule products by thin-layer chromatography and ultra-performance liquid chromatography
Source: Chin Med. 2015 Jun 6;10:13. doi: 10.1186/s13020-015-0040-2 (PMC4477300; doi:10.1186/s13020-015-0040-2)
Supplement: Additional file 3: — TLC of methanol extract and residue from the raw herb/granule. Lane 1: Methanol extract of granule sample 4 (G4); Lane 2: Residue of the methanol extract of G4; Lane 3: Water and methanol extract of the raw herb R1; Lane 4: Residue of water and methanol extract of raw herb. As shown in the figure, Lanes 1 and 3 of the methanol extract contain many bands which represented the marker compounds, whereas Lanes 2 and 4 were absent of the bands. This supported the assumption that methanol efficiently extracted the major compounds. [file 13020_2015_40_MOESM3_ESM.pdf]

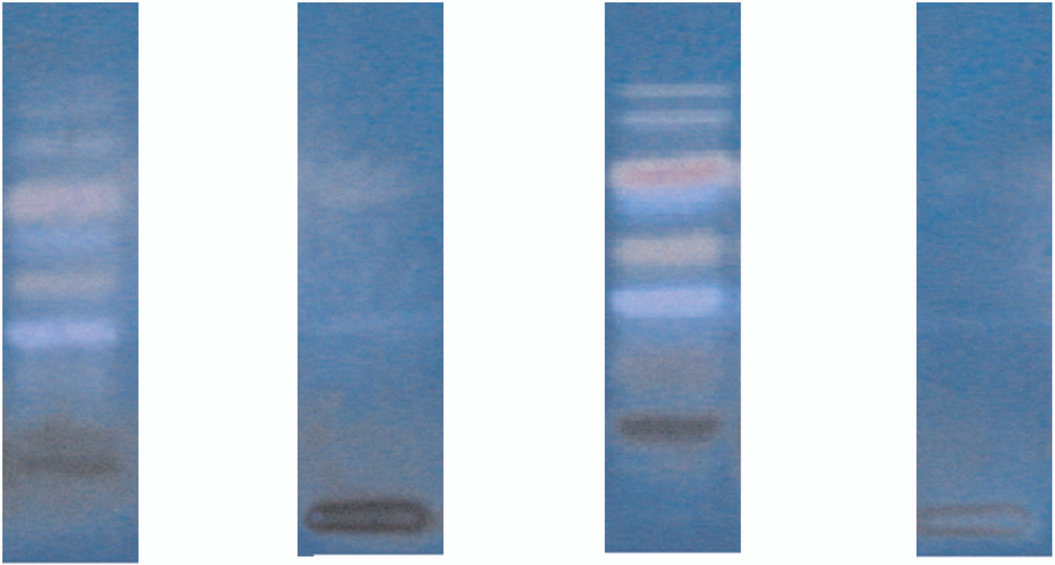

Lane 1
Methanol extract of G4

Lane 2
Residue of the methanol extract of G4

Lane 3
Water and methanol extract of R1

Lane 4
Residue of water and methanol extract of R1
