# Supplementary figures and images for: A multivariate analysis on the comparison of raw notoginseng (Sanqi) and its granule products by thin-layer chromatography and ultra-performance liquid chromatography
Source: Chin Med. 2015 Jun 6;10:13. doi: 10.1186/s13020-015-0040-2 (PMC4477300; doi:10.1186/s13020-015-0040-2)

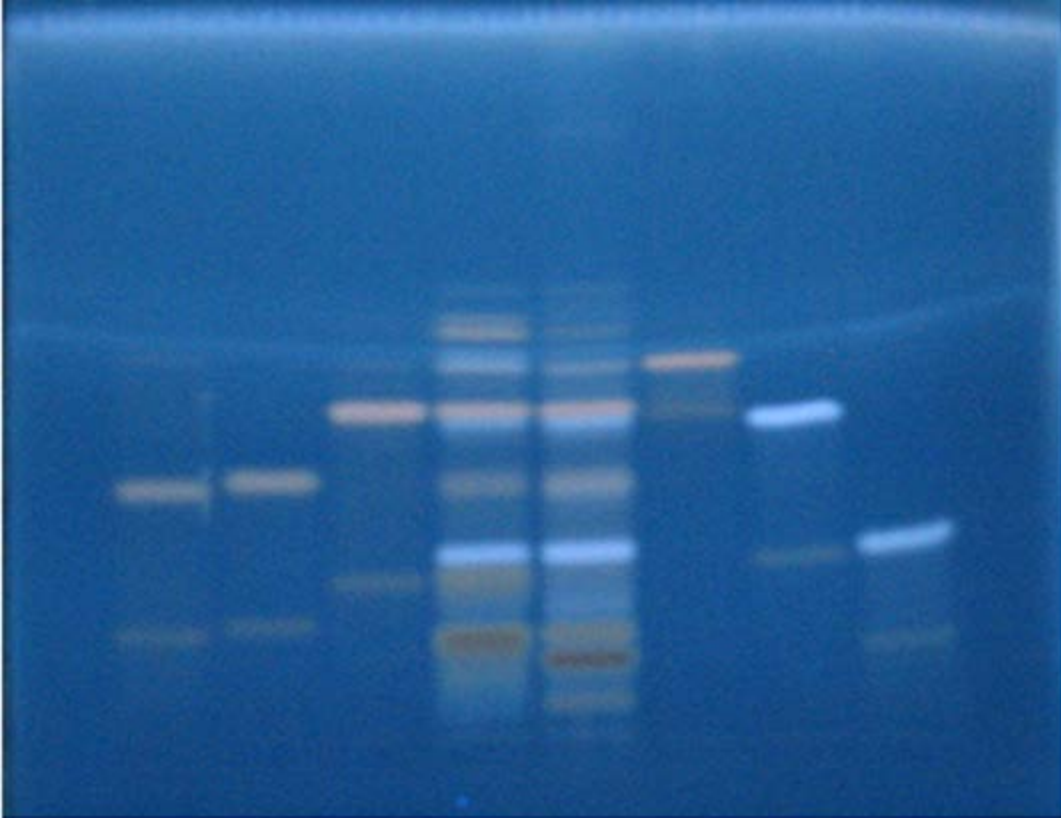

1

2

3

4

5

6

7

8

Supplement: Additional file 4: — TLC fluorescence image of Sanqi raw herb and granule samples with the standards under UV mode (366 nm). Key: Lane 1 – Notoginseng NR1; Lane 2 – Ginsenoside Re; Lane 3 – Ginsenoside Rg1; Lane 4 - R6 methanol extract; Lane 5 - G12 methanol extract; Lane 6 -Ginsenoside Rg2; Lane 7 – Ginsenoside Rd; Lane 8– Ginsenoside Rb1. [file 13020_2015_40_MOESM4_ESM.pdf]

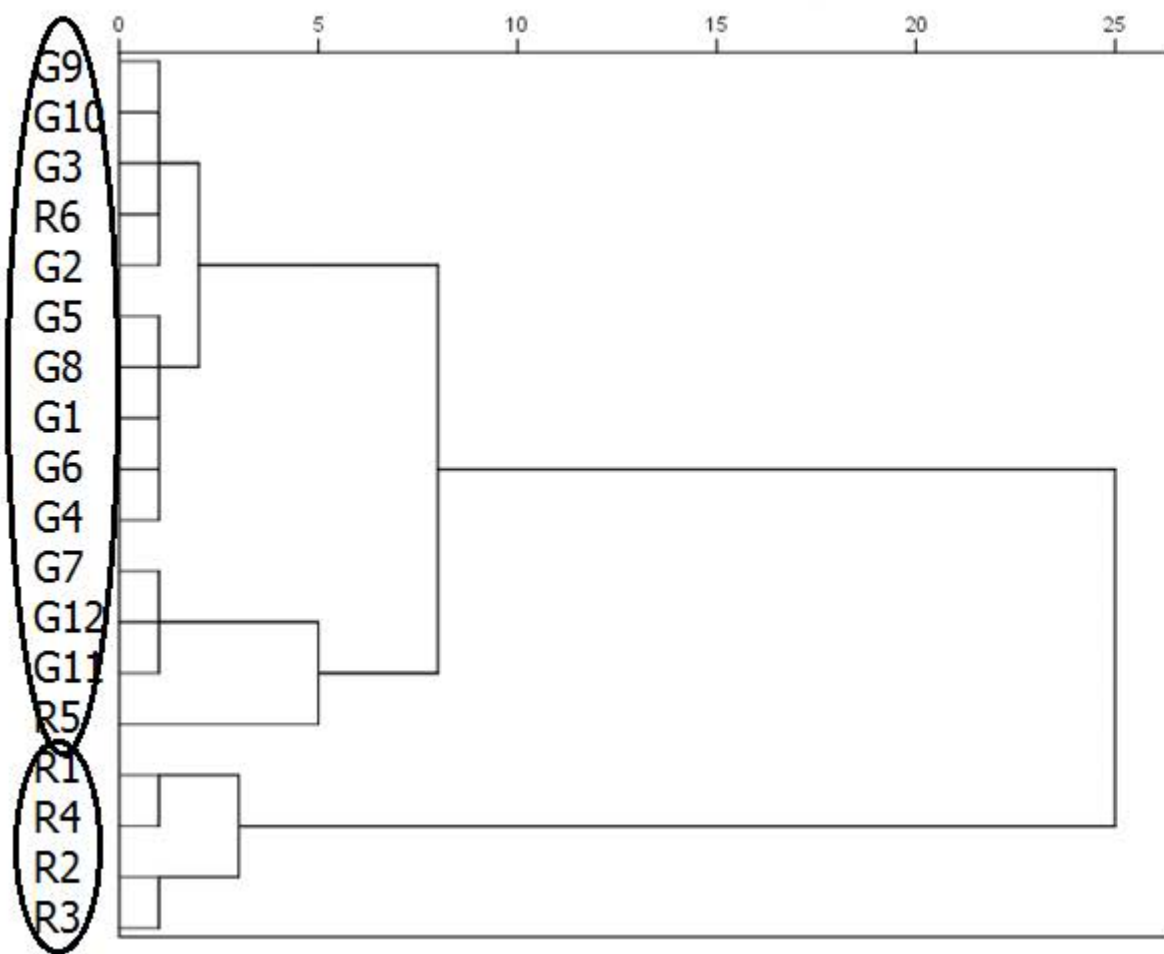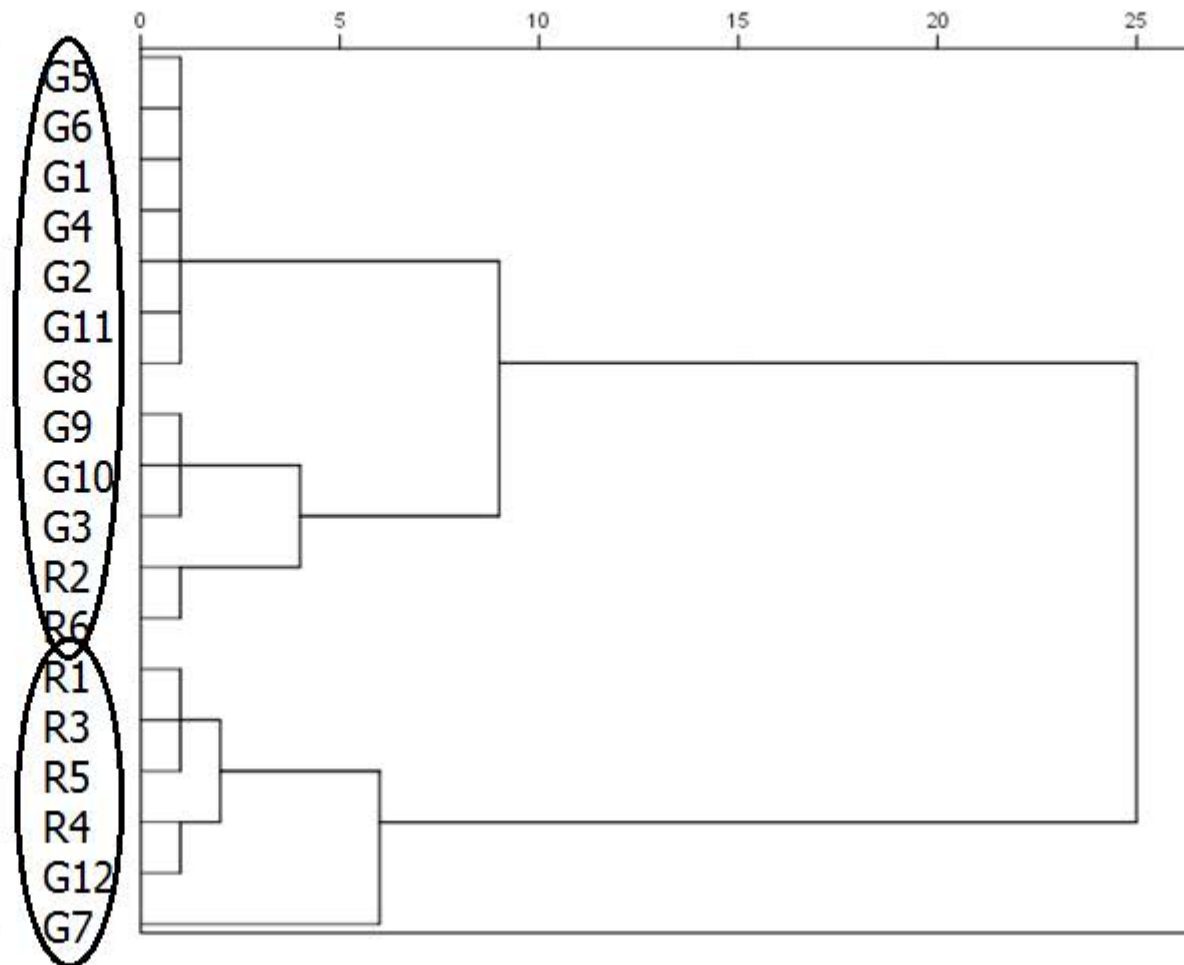

Supplement: Additional file 5: — HCA dendograms of Sanqi extracts analysed by (A) TLC and (B) UPLC results using three markers (NR1, Rg1 and Rb1). For TLC, HCA revealed that the clusters were identical to those derived from the content of the five marker compounds. However, for the HCA result analysed by UPLC, R2 and R6 were grouped with the granules. [file 13020_2015_40_MOESM5_ESM.pdf]
